# Combined Association of Body Mass Index and Alcohol Consumption With Biomarkers for Liver Injury and Incidence of Liver Disease: A Mendelian Randomization Study

**DOI:** 10.1001/jamanetworkopen.2019.0305

**Published:** 2019-03-08

**Authors:** Alice R. Carter, Maria-Carolina Borges, Marianne Benn, Anne Tybjærg-Hansen, George Davey Smith, Børge G. Nordestgaard, Debbie A. Lawlor

**Affiliations:** 1 MRC Integrative Epidemiology Unit, University of Bristol, Bristol, United Kingdom; 2 Population Health Science, Bristol Medical School, University of Bristol, United Kingdom; 3 Department of Clinical Biochemistry, Rigshospitalet, Copenhagen, Denmark; 4 The Copenhagen General Population Study, Herlev and Gentofte Hospital, Copenhagen, Denmark; 5 Copenhagen University Hospital, Faculty of Health and Medical Sciences, University of Copenhagen, Copenhagen, Denmark; 6 Department of Clinical Biochemistry, Herlev and Gentofte Hospital, Copenhagen, Denmark

## Abstract

**Importance:**

Individually, higher body mass index (BMI) and alcohol consumption increase the risk of liver disease. Evidence of a joint association is mixed; however, previous studies have not used causal inference methods robust to confounding and reverse causation. Understanding any true effect is key to developing effective interventions to reduce liver disease.

**Objective:**

To investigate the joint association of BMI and alcohol consumption with liver injury biomarkers and incident liver disease using factorial mendelian randomization (MR).

**Design, Setting, and Participants:**

A population-based cohort study (Copenhagen General Population Study) recruited a random sample of Copenhagen, Denmark, residents aged 20 years or older of white, Danish descent (N = 98 643) between November 25, 2003, and July 1, 2014. Data were also obtained from ongoing links to national registers, and then analyzed from September 30, 2016, to April 23, 2018.

**Exposures:**

High and low BMI and alcohol consumption categories from baseline-measured or self-reported observational data and genetic variants predicting BMI and alcohol consumption.

**Main Outcomes and Measures:**

Plasma biomarkers of liver injury (alanine aminotransferase [ALT] and γ-glutamyltransferase [GGT]) and incident cases of liver disease from hospital records were the outcomes.

**Results:**

Of the 98 643 individuals recruited, 91 552 (54 299 [45.2%] women; mean [SD] age, 58 [13.05] years) with no baseline liver disease were included in main analyses. Individuals had a mean (SD) BMI of 26.2 (4.3) and consumed a mean (SD) of 10.6 (10.2) U/wk of alcohol. In factorial MR analyses, considering the high BMI/high alcohol group as the reference, mean circulating ALT and GGT levels were lowest in the low BMI/low alcohol group (ALT: −2.32%; 95% CI, −4.29% to −0.35%, and GGT: −3.56%; 95% CI, −5.88% to −1.24%). Individuals with low BMI/high alcohol use and high BMI/low alcohol use also had lower mean circulating ALT levels (low BMI/high alcohol use: −1.31%; 95% CI, −1.88% to −0.73%, and high BMI/low alcohol use: −0.81%; 95% CI, −2.86% to 1.22%) and GGT levels (low BMI/high alcohol use: −0.91%; 95% CI, −1.60% to −0.22%, and high BMI/low alcohol use: −1.13%; 95% CI, −3.55% to 1.30%) compared with the high BMI/high alcohol use reference group. These patterns were similar in multivariable factorial analyses. For incident liver disease (N = 580), factorial MR results were less conclusive (odds ratio of liver disease vs high BMI/high alcohol group: 1.01; 95% CI, 0.84 to 1.18, for the low BMI/high alcohol group, 1.22; 95% CI, 0.56 to 1.88 for the high BMI/low alcohol group, and 0.99 (95% CI, 0.41 to 1.56 for the low BMI/low alcohol group).

**Conclusions and Relevance:**

Interventions to reduce both BMI and alcohol consumption might reduce population levels of biomarkers of liver injury more than interventions aimed at either BMI or alcohol use alone. However, it is not clear whether this intervention will directly translate to a reduced risk of liver disease.

## Key Points

**Question:**

Is there a joint association of body mass index and alcohol consumption with liver disease and liver injury biomarkers?

**Findings:**

In this mendelian randomization study of a population-based cohort of 91 552 European adults, compared with individuals categorized as having both a high body mass index and weekly units of alcohol consumption, those in the low category for both of these risk factors were associated with lower circulating liver injury biomarkers compared with being high for either one or both of the risk factors. However, this association was less clear when considering cases of liver disease as the outcome.

**Meaning:**

Interventions to reduce both body mass index and alcohol consumption may result in the greatest reduction in circulating liver injury biomarkers, but these results do not appear to be able to demonstrate whether these interventions will result in a reduction in cases of liver disease.

## Teaser

This population-based cohort study uses mendelian randomization to examine the association of body mass index (BMI) and alcohol consumption with levels of liver injury biomarkers and incident liver disease in adults in Denmark.

## Introduction

The risk of liver disease has increased, and the age at first diagnosis has decreased in many high-income countries in recent decades.^1^ A key contributor is likely to be the global obesity epidemic and the association between greater adiposity and nonalcoholic fatty liver disease.^2^ Increasing or sustained unhealthy levels of alcohol consumption in some countries are also likely to have contributed to the increase.^3^

Laboratory-based studies have suggested that shared biochemical pathways exist between adiposity and alcohol leading to liver disease, including via insulin resistant pathways.^4^ Epidemiologic studies suggest that overweight and alcohol consumption positively interact on a multiplicative scale to increase the risk of pathologic liver changes, suggesting the relative risk of liver disease in people who drink excessively and are overweight or obese is greater than would be expected if the 2 risk factors were independent.^5^ Consistent with these findings, several studies have found that greater body mass index (BMI) and alcohol consumption positively interact in association with biomarkers of liver injury, such as alanine aminotransferase (ALT), aspartate aminotransferase, and γ-glutamyltransferase (GGT).^6,7^ By contrast, in a study of British women (approximately 1.2 million women with 1811 occurrences of a first hospital admission or death from liver cirrhosis), there was no evidence of an interaction between BMI and alcohol associated with cirrhosis.^8^

**Figure 1.**
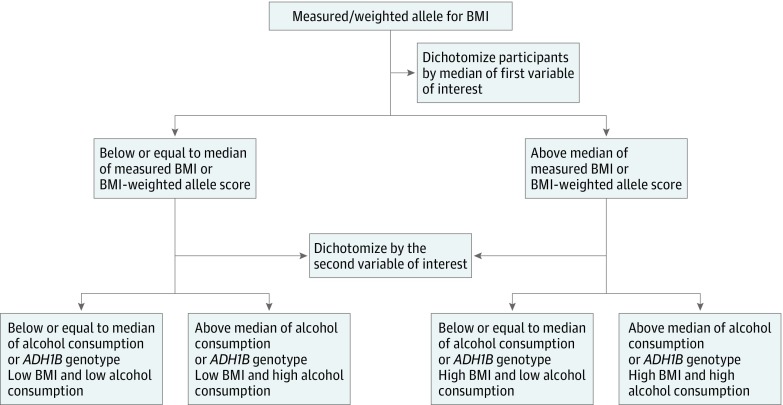
Flow Diagram for the Generation of Factorial Groups, Using Observational and Genetic Data Factorial multivariable: both body mass index (BMI) and weekly alcohol consumption were dichotomized based on the median of measured or self-reported values. Values equal to or below the median were categorized as the low group, and those above the median were categorized as the high group. Factorial mendelian randomization (MR): For genetic propensity, BMI was categorized according to the median of the weighted allele score for BMI, with values equal to or below the median categorized as low BMI and those above the median categorized as high BMI. Alcohol propensity was determined according to *ADH1B* alleles. Individuals who were homozygous for the alcohol-decreasing traits and heterozygous individuals were combined, as determined to be appropriate based on previous MR analyses of these traits on alcohol intake,^11^ to create the low alcohol-propensity group. The high alcohol-propensity group contains all individuals homozygous for the alcohol-increasing trait. Adapted from Ference et al.^12^

Understanding the combined effects of BMI and alcohol consumption on liver disease is important for developing preventive public health interventions and establishing the likely future burden of liver disease in populations with differing levels of high BMI and high alcohol consumption. Previous findings from observational studies might be explained by residual confounding, misclassification bias, or reverse causality. Mendelian randomization (MR), the use of genetic variants as instrumental variables for assessing the effect of modifiable exposures, is increasingly used to infer causal relationships between risk factors and outcomes. Some of the key strengths of MR include being more robust to reverse causality, confounding, and measurement error than traditional observational multivariable epidemiologic studies.^9,10^ These biases are important when considering an exposure such as alcohol consumption, which is typically poorly reported by individuals and is influenced by multiple socioeconomic, lifestyle, and health factors. Mendelian randomization can be used in a factorial design when considering how 2 or more risk factors may work together to influence an outcome, comparable to a factorial, randomized clinical trial design (Figure 1, adapted from Ference et al^12^).

The aim of this study was to use observational regression and factorial MR analyses to investigate the joint association of BMI and alcohol consumption with liver injury biomarkers (ALT and GGT) and liver disease.

## Methods

We used data from the Copenhagen General Population Study (N = 98 643), a large population cohort with genotypic and phenotypic data on a wide range of health-related problems. Copenhagen, Denmark, residents 20 years or older and of white, Danish descent were randomly recruited from the national Danish Civil Registration between November 25, 2003, and July 1, 2014. Data were also obtained from ongoing links to national registers and then analyzed from September 30, 2016, to April 23, 2018. Ethics approval for the study was obtained from Herlev Hospital and the Danish ethical committee, and all participants provided written informed consent. Participants did not receive financial compensation. Additional study details have been published.^13^ This study followed the Strengthening the Reporting of Observational Studies in Epidemiology (STROBE) reporting guideline.

**Figure 2.**
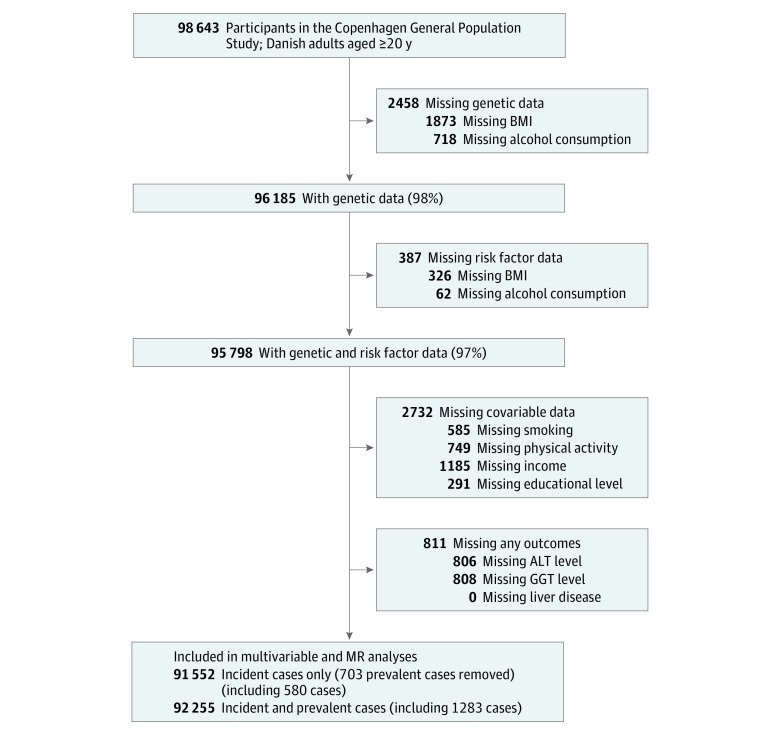
Missing Data and Total Number of Participants for Each Analysis Participants could have had missing data for more than 1 variable at each stage; hence, the additive numbers missing for each variable can total more than the number removed at that stage. ALT indicates alanine aminotransferase; BMI, body mass index; GGT, γ-glutamyltransferase; MR, mendelian randomization.

Genetic data were available on 96 185 participants (98%). Body mass index and alcohol data were missing for a further 387 individuals, leaving 95 798 participants (97%) available for analysis. There were some further missing data for additional covariables (smoking, physical activity, income, and educational level) in 2732 individuals, who were subsequently excluded from analyses. A diagnosis of liver disease is likely to result in treatment and lifestyle changes; therefore, individuals with known liver disease (as defined by *International Classification of Diseases [ICD]* codes listed in eTable 1 in the Supplement and described below) at baseline (prevalent cases) were excluded from our main analyses (Figure 2).

All measurements were completed by trained staff at 1 clinic center. Weight was measured without shoes and in light clothing to the nearest 0.1 kg (Soehnle Professional scales). Height was measured to the nearest 0.1 cm (seca stadiometer). Usual alcohol intake was reported as weekly consumption of beer in bottles and standard glasses of wine and spirits. Each of these products in Denmark contains 1 U of alcohol or the equivalent of approximately 12 g of pure alcohol.

We used 5 genetic variants that were available in the database and are associated with BMI and fat mass to create a weighted allele score as an instrumental variable for BMI: *FTO* rs9939609, *TMEM18* rs6548238, *MC4R* rs17782313, *BDNF* rs10767664, and *GNPDA2* rs6548238. In the absence of genome-wide data, these variants were chosen for genotyping because they have the largest known common effect sizes for the association with BMI in European populations.^14^ This score was externally weighted according to the β value for each genetic variant association with BMI from the most recent genome-wide association study.^14^ A single genetic variant, *ADH1B* (rs1229984, Arg47His in exon 3), was used as an instrumental variable for alcohol consumption. This variant is associated with alcohol consumption, as demonstrated in previous analyses using these data.^11,13^ Genotyping was conducted by laboratory technicians without access to participant data (ABI PRISM 7900HT Sequence Detection System; Applied Biosystems Inc) and TaqMan assays. Genotyping was verified by DNA sequence in at least 30 individuals with each genotype. Reruns were performed twice and 99.96% of all available participants were genotyped.

Nonfasting plasma ALT and GGT levels were measured at baseline using standard hospital assays (Konelab, Thermo Fischer Scientific, Denmark, and ACL Top blood coagulation analyzer, ILS Denmark) and were subject to daily internal quality control assessing assay precision and monthly external quality control assessing assay accuracy. Participants were linked to their hospital records providing information on all admissions and outpatient appointments before and after study recruitment, with diagnoses made according to routine clinical care. These hospital records identified individuals with existing chronic liver disease and those who developed liver disease during follow-up (incident cases). Participants were also linked to death records. The following *ICD* diagnostic terms, incorporating both alcoholic and nonalcoholic modifiable causes, were included as cases of chronic liver disease: cirrhosis, including alcoholic fatty liver; unspecified chronic liver disease without mention of alcohol; hepatic fibrosis; malignant neoplasms of the liver; acute and subacute necrosis of the liver; chronic passive congestion of the liver; unspecified disorder of the liver; abdominal swelling; and other nonspecific liver disease.^15^ eTable 1 in the Supplement provides the *ICD* codes used.

Participants completed a questionnaire that recorded details on smoking (never vs ever smoker), physical activity (low, 0-4 hours per week vs high, >4 hours per week), income (low/middle income, ≤600.000kr [$66 348 US$] per year vs high income, >600.000kr per year), and educational attainment (low/middle education, ≤13 years of schooling vs high education >13 years), which were included as covariables.

### Statistical Analysis

We explored the separate and joint associations of BMI and alcohol consumption with ALT, GGT, and liver disease using both observational multivariable and MR analyses. In multivariable and MR analyses, the results reflect the same magnitude of change in exposures (ie, 1-SD score higher BMI or 1-U greater alcohol intake on outcome). Alanine aminotransferase and GGT levels were natural log-transformed to normalize the distribution of residuals in regression models. Body mass index was standardized for age (5-year groups) and sex into SD scores. Both the multivariable analysis and MR analysis are on a multiplicative scale, and results for biomarkers are presented as percentage differences in mean levels per 1 BMI SD or 1 U of alcohol. All analyses were conducted in Stata, version 15 (StataCorp).

#### Multivariable Analyses

In multivariable analyses, we examined BMI in categories of normal weight or underweight (<25.0 [calculated as weight in kilograms divided by height in meters squared]), overweight (25.0-29.9), and obese (≥30.0), and as a continuous BMI SD score. We examined alcohol use in categories of none, 1 to 14, and 15 U or more per week, and per unit of alcohol consumption per week. We used multivariable linear regression to examine associations with ALT and GGT levels and logistic regression for associations with liver disease.

To examine joint associations of BMI and alcohol consumption with markers of liver injury, a factorial analysis was completed by categorizing participants in 4 groups according to their measured values of the exposure: high BMI/high alcohol consumption (reference), high BMI/low alcohol consumption, high alcohol consumption/low BMI, and low BMI/low alcohol consumption. Low and high BMI were defined as less than or equal to or greater than median BMI SD score, respectively. Similarly, low and high alcohol consumption were defined as less than or equal to or greater than median alcohol consumption, respectively. The mean difference between the high and low groups was 1.49 per SD of BMI and 14.68 U/wk for alcohol consumption (Figure 1). Multivariable linear or logistic regression was then used to assess the association of each of these groups with outcomes. Stratified analyses of BMI SD score with each outcome by strata of alcohol consumption (none, <15, and ≥15 U/wk) were also performed. A likelihood ratio test was used to test for statistical evidence of interaction by comparing a model with BMI SD score and alcohol categories independent with one where an interaction term was included.

For all multivariable analyses, we considered the best estimate of an effect to be adjusted for all observed variables that we considered potential confounders (age, sex, cigarette smoking, physical activity, educational attainment, and income) and individuals with baseline liver disease removed.

#### MR Analyses

An exact test was used to examine Hardy-Weinberg equilibrium of genotype frequencies.^16^ We present the first-stage (regression of risk factor on genetic instrument) *F* statistic and *R*^2^ as a measure of the association between genetic instruments and observed phenotype. We measured associations of BMI and alcohol consumption instruments with observed confounders to test our a priori assumption of no confounding associations. Mendelian randomization analyses were completed using the 2-stage least squares instrumental variable regression method for continuous outcomes. For the binary outcome (liver disease), MR was run in 2 regression stages, including robust SEs in the second stage.^17,18^

As with multivariable analyses, a factorial analysis was completed to test joint associations, categorizing participants to the same 4 groups previously described according to genetic propensities. Low and high BMI were defined as less than or equal to or greater than median BMI-weighted allele score, respectively. Alcohol consumption was dichotomized according to *ADH1B* alleles, where individuals homozygous for the alcohol-decreasing allele or heterozygous for *ADH1B* were classed as low alcohol consumption, and the high alcohol consumption group comprised individuals homozygous for the alcohol-increasing allele of *ADH1B*^11^ (Figure 1). The mean difference between the high and low groups was 0.51 BMI SD and 1.78 U/wk for alcohol consumption (Figure 1). Regression methods were then used to assess the association of each of these groups with outcomes. We investigated evidence of an interaction between BMI and alcohol consumption using stratified MR of the BMI-weighted allele score instrument by self-reported weekly alcohol consumption (none, <15, and ≥15 U/wk). A variance-weighted, least squares approach was used to test for statistical evidence of a difference in circulating biomarkers and liver disease risk across alcohol strata. This evaluation was done by calculating a χ^2^ test to assess for deviations between point estimates in each stratum.

#### Comparing Multivariable and MR Results

We compared the multivariable and MR results by using a χ^2^ test to examine the null hypothesis that the coefficients are consistent with one another. We made this comparison for analyses of the association of BMI and alcohol separately, and for the stratified results of the association of 1-SD greater BMI within the strata of reported alcohol consumption. We were not able to compare the magnitude of effect sizes of the multivariable and MR factorial analyses because it was not possible to split participants into high and low BMI and alcohol groups in identical ways for the 2 methods. Thus, in the multivariable analyses, the high vs low results reflect a 1.49-SD difference in BMI and a 14.68-U difference in units of weekly alcohol consumption, whereas the equivalent differences in the MR analyses are 0.51 SD and 1.78 U/wk, respectively. We did, however, compare the directions and overall patterns of association for the factorial results.

### Sensitivity Analyses

Sensitivity analyses were completed to test the robustness of our MR results for the associations of BMI on outcomes. These analyses included using methods that are more robust to pleiotropic variants (ie, weighted median methods and MR-Egger method),^19,20^ and assessing the association of outlying variants by removing 1 variant at a time and recalculating the overall MR estimate (ie, leave-1-out analysis). Because we have only 1 genetic variant for alcohol, these sensitivity analyses were not possible. For MR analyses of both BMI and alcohol consumption, we adjusted for income and smoking because of some evidence of genetic instrument associations with these variables as a sensitivity analysis. All analyses were repeated with individuals with prevalent cases of liver disease included.

## Results

Of the 98 643 participants, 91 552 (54 299 [45.2%] women; mean [SD] age, 58 [13.05] years) with no baseline liver disease were included in main analyses. A total of 9.4% of the participants reported not drinking alcohol and 12.3% reported drinking 22 U/wk or more (eTable 2 in the Supplement). Both alcohol intake and BMI differed by sex (eTable 2 in the Supplement). In women, 12.4% reported not drinking alcohol compared with 5.8% of men. Conversely only 0.8% of women reported drinking more than 22 U per week, compared with 5.8% of men. A total of 42.9% of the participants were normal weight; 0.7%, underweight; 40.2%, overweight; and 16.3%, obese. There was a greater proportion of women who were normal weight (52.3%) compared with men (32.7%), and a greater proportion of overweight men (49.7%) compared with women (32.4%) (eTable 2 in the Supplement). The sex distribution was equal in the high or low genetic factorial groups, as would be expected given the assumption of no confounding in MR, but differences were present in the observational factorial groups (eTable 3 in the Supplement).

There were 1405 cases (1.4%) of either prevalent or incident liver disease, with 616 (0.6%) incident cases. Of these cases, there were 420 prevalent cases of alcohol-induced liver disease and 356 incident cases of alcohol-induced liver disease (eTable 2 in the Supplement). All measured confounders were associated with reported alcohol intake and measured BMI (eTable 4 in the Supplement). For example, compared with never smokers, current or former smokers had an increased BMI of 0.36 (95% CI, 0.30-0.41) and an increased alcohol consumption of 2.90 U/wk (95% CI, 2.77-3.03 U/wk), but most were not associated with *ADH1B* or the BMI-weighted allele score (eTable 5 in the Supplement). There was some evidence that the BMI-weighted allele score was positively associated with smoking and negatively associated with income, where the BMI-weighted allele score was increased by 0.002 U (95% CI, 0.0006-0.003 U) for current or former smokers compared with never smokers,. All observed confounders were associated with the factorial groups in multivariable analysis; none were associated with factorial groups in MR analysis (eTable 6 in the Supplement). The minor allele frequencies for all 5 genotypes are reported in eTable 7 in the Supplement; all were in Hardy-Weinberg equilibrium. The first-stage partial *F* statistic and *R*^2^ for the combined weighted allele score for BMI were 453 and 0.0049, and for *ADH1B* were 117 and 0.0013, respectively (eTable 8 in the Supplement).

**Figure 3.**
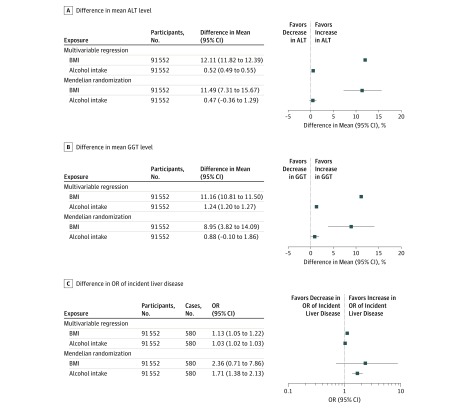
Multivariable and Mendelian Randomization Analyses of the Individual Associations of Body Mass Index (BMI) and Alcohol Consumption With Liver Enzyme Levels Associated With Injury and Incident Liver Disease A, The percentage difference in mean alanine aminotransferase (ALT) level per 1-SD higher BMI or per 1 U/wk increase in alcohol consumption. B, The percentage difference in mean γ-glutamyltransferase (GGT) level per 1-SD higher BMI or per 1-U increase in alcohol consumption. C, The difference in odds ratio (OR) of incident liver disease per 1-SD higher BMI or per 1-U/wk increase in alcohol consumption. The BMI is measured as age and sex SD units. Alcohol measured as units of alcohol consumed per week, where 1 U is equivalent to 12 g of alcohol. Multivariable analyses were adjusted for age, sex, smoking, educational level, income, and physical activity. Mendelian randomization used weighted allele score for BMI and *ADH1B* alleles for alcohol consumption. Prevalent cases of liver disease were excluded from all analyses.

In both multivariable and MR analyses, individually higher BMI and higher alcohol consumption were associated with higher mean ALT and GGT levels and odds of incident liver disease (Figure 3). Overall, multivariable and MR results were consistent with each other, although point estimates for ALT and GGT levels appeared slightly larger in multivariable compared with MR analyses (Figure 3A and B). For both methods, the associations with ALT and GGT levels were similar for BMI, whereas the association of alcohol consumption with GGT was more than double the magnitude of the association of alcohol with ALT levels, although in both cases the associations are small and the 95% CIs overlap.

Larger associations of both BMI and alcohol consumption were seen in the MR analysis compared with multivariable regression for incident liver disease. However, the 95% CIs in the MR analyses were wide and included the null value, particularly in the case of BMI (Figure 3C). Analysis including individuals with prevalent cases of liver disease showed similar estimates of associations with analyses restricted to incident cases (eFigure 1 and eFigure 2 in the Supplement).

Multivariable analysis indicated a linear association of increasing BMI and increasing alcohol consumption with increasing levels of biomarkers for liver injury and odds of disease (eFigure 3 and eFigure 4 in the Supplement); nonlinear associations were not tested in MR analyses.

**Figure 4.**
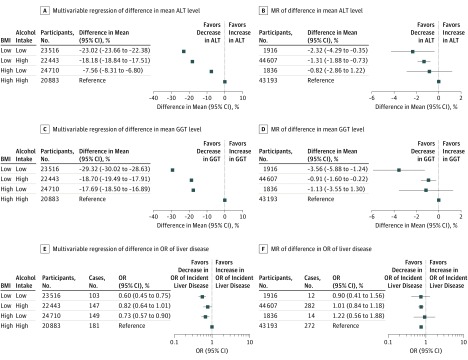
Multivariable and Mendelian Randomization (MR) Factorial Analyses Assessing the Joint Associations of Body Mass Index (BMI) and Alcohol With Biomarkers of Liver Injury and Incident Liver Disease A, Multivariable regression of percentage difference in mean alanine aminotransferase (ALT) level by joint observational BMI and alcohol categories. B, MR of percentage difference in mean ALT level by joint genetic BMI and alcohol categories. C, Multivariable regression of percentage difference in mean γ-glutamyltransferase (GGT) level by joint observational BMI and alcohol categories. D, MR of percentage difference in mean GGT level by joint genetic BMI and alcohol categories. E, Multivariable regression of odds ratio (OR) of liver disease by joint observational BMI and alcohol categories. F, MR of OR of liver disease by joint genetic BMI and alcohol categories. Low vs high BMI (calculated as weight in kilograms divided by height in meters squared), 1.49-SD difference in multivariable analyses and 0.51 SD in MR analyses. Low vs high alcohol, 14.68 U/wk difference in multivariable analyses and 1.78 U/wk difference in MR analyses, where 1 U of alcohol is equivalent to 12 g. Multivariable analysis was adjusted for age, sex, smoking, educational level, income, and physical activity.

In both multivariable and MR factorial analyses, patients in the low BMI/low alcohol consumption groups had the lowest mean ALT and GGT levels in comparison with those in the highest categories of both biomarkers. In factorial MR analyses, mean circulating ALT and GGT were reduced by −2.32% (95% CI, −4.29% to −0.35%) and −3.56% (95% CI, −5.88% to −1.24%), respectively. Individuals with low BMI/high alcohol use and high BMI/low alcohol use also had lower mean circulating ALT levels (low BMI/high alcohol use: −1.31%; 95% CI, −1.88% to −0.73% and high BMI/low alcohol use: −0.81%; 95% CI, −2.86% to 1.22%, respectively) and GGT levels (low BMI/high alcohol use: −0.91%; 95% CI, −1.60% to −0.22% and high BMI/low alcohol use: and −1.13%; 95% CI, −3.55% to 1.30%) compared with the high BMI/high alcohol use reference group. These patterns were similar in multivariable factorial analyses (Figure 4A-D). In both sets of analyses, being in the low BMI/high alcohol consumption group conferred a greater protective association with ALT levels than those in the high BMI/low alcohol consumption group compared with the reference group. However, for GGT levels, being low for either BMI or alcohol consumption had almost identical protective associations compared with the reference group, in both multivariable and MR analyses. These results for liver biomarkers were similar when individuals with prevalent cases of liver disease were included in the analysis (eFigure 5 in the Supplement).

For the odds of incident liver disease, factorial multivariable regression analyses had a pattern similar to that of the biomarkers (ie, reduced odds in those with both low BMI and alcohol intake, in comparison with those high for both risk factors, and in between for the 2 mixed groups) (Figure 4E and F). This pattern was not consistent in the factorial MR analyses. These patterns were similar in multivariable factorial analyses. For incident liver disease (N = 580), factorial MR results were less conclusive (odds ratio of liver disease vs high BMI/high alcohol group: 1.01; 95% CI, 0.84 to 1.18, for the low BMI/high alcohol group, 1.22; 95% CI, 0.56 to 1.88 for the high BMI/low alcohol group, and 0.99 (95% CI, 0.41 to 1.56 for the low BMI/low alcohol group). When analyses were repeated with both incident and prevalent liver disease included as the outcome, factorial multivariable analyses suggested that patients with low BMI/high alcohol intake had reduced odds similar to those with low values of both risk factors, which was not confirmed by factorial MR (eFigure 6 in the Supplement).

In stratified multivariable analyses, the positive association of BMI with all outcomes was strongest in patients consuming 15 U or more of alcohol per week compared with those not drinking alcohol or drinking less than 15 U per week. There was statistical support for a positive interaction on a multiplicative scale between the 2 risk factors for ALT and GGT levels; this evidence was weaker for the odds of liver disease, but the pattern of interaction was similar (eFigure 7 in the Supplement). The same pattern of positive multiplicative interaction of BMI and alcohol consumption was not observed for any of the outcomes in MR analyses (eFigure 7 in the Supplement). For ALT and GGT levels, the MR results suggested a weaker positive association of BMI in those with highest alcohol consumption (≥15 U/wk), although these associations were imprecisely estimated, and we found no strong evidence for any interaction. For liver disease, the stratified MR analyses were particularly imprecise, although again results suggested, if anything, a negative interaction.

### Sensitivity Analyses

Weighted median MR and MR-Egger estimates for the association of BMI with outcomes were broadly similar to our main MR analyses results (eTable 9 in the Supplement). Heterogeneity between the 5 BMI-related genetic variants used as instrumental variables in MR was modest (*I*^2^ = 52% for ALT level, 13% for GGT level, and 38% for the odds of incident liver disease). Results were consistent when 1 genetic variant at a time was removed, although for both ALT and GGT levels, the estimates were attenuated slightly when *FTO* was removed (eFigure 8 in the Supplement). Adjustment of the MR analyses for smoking and income did not notably influence the results.

## Discussion

Using both multivariable and MR analyses, we have shown that higher BMI and alcohol consumption are associated with higher circulating levels of ALT and GGT individually and jointly and may infer a causal relationship. The broad consistency of findings between multivariable and MR analyses for each exposure individually, and both jointly, on ALT and GGT provides more compelling evidence for potential causality than either method alone as they each have different key sources of bias.^21^ Mendelian randomization findings for incident liver disease suggested larger estimates of an association with both BMI and alcohol intake than the observational multivariable findings in multivariable analyses or horizontal pleiotropy exaggerating associations in MR analyses. However, these estimates were imprecise and the wide 95% CIs for BMI included the null value.

Consistent with individual associations, multivariable and MR factorial analyses suggested that adults with low BMI/low alcohol consumption had lower ALT and GGT levels compared with those with high levels of both risk factors. Patients with low levels of 1 risk factor had intermediate levels of ALT and GGT. The higher concentrations of ALT and GGT in the high BMI/high alcohol intake group could reflect the combination of independent associations of BMI and alcohol intake or a positive interaction between the 2 risk factors. We found some evidence of positive multiplicative interactions of BMI and alcohol intake with ALT and GGT levels in multivariable analyses, but not in MR analyses. For incident liver disease, the multivariable factorial and stratified analyses followed the same pattern of those seen with ALT and GGT levels, whereas in MR analyses there was no clear evidence of differences across factorial groups. However, 95% CIs for factorial or interaction analyses for incident liver disease were wide, suggesting a lack of statistical power despite the large sample size.

Our multivariable analyses are consistent with most previously published studies of the individual associations of BMI and alcohol intake on liver biomarkers and disease.^5,11,15^ Using MR in a smaller number of participants from the same study population as used here (N = 58 313), a possible causal association of greater alcohol consumption on ALT and GGT levels was shown.^11^ We have added to that previous work by estimating the associations of increasing BMI with ALT and GGT levels as well as an incident liver disease and undertaking the first, to our knowledge, MR analyses of the joint associations of BMI and alcohol intake with these outcomes. Consistent with our multivariable analyses, some,^6,7^ although not all,^8^ previous multivariable studies reported a positive multiplicative interaction between higher BMI and alcohol consumption with liver biomarkers and disease. However, our MR analyses suggest that BMI and alcohol intake combine as we would expect if their individual associations with ALT and GGT levels are independent of each other, as we observed a nonlinear pattern in stratified MR, and possibly with smaller associations than independence for liver disease, although power would have been low. In the absence of other MR studies of these joint associations, we suggest caution regarding the joint associations with liver disease outcomes.

### Strengths and Limitations

Our study has several strengths, including the large sample size, the assessment of potential single and joint associations of BMI and alcohol intake with liver biomarker and disease outcomes using both multivariable and MR, and appropriate sensitivity analyses to test the robustness of the MR assumptions. To our knowledge, because they are relatively rare, incident cases of liver disease have not been explored previously with MR or in many multivariable regression studies. The study of liver biomarkers, high levels of which reflect cell damage in the liver and biliary track, as well as disease outcomes, is valuable for indicating propensity to future disease and allowing analyses with greater statistical power.

The study also has limitations. Mendelian randomization and multivariable analyses are potentially biased by violation of their assumptions. Multivariable approaches assume that all confounders have been accurately measured and accounted for, and that there is no reverse causation, selection bias, or systematic measurement error that distorts the estimate.^21^ The removal of patients with prevalent liver disease in our main analyses should have limited potential survivor bias and reverse causality. We adjusted for key observed potential confounders, but residual confounding by unmeasured confounders or imprecise measurement might bias estimates in our multivariable analyses. For the individual associations of BMI and alcohol use with ALT and GGT levels as well as liver disease, multivariable results were similar to the MR results, which are less prone to confounding, suggesting that residual confounding might not be a major issue.^22^ However, the larger individual associations in MR analyses of liver disease might mean that multivariable results were biased by masking residual confounding.

Mendelian randomization analyses assume that there is a robust association between the genetic instrument and risk factor, that the genetic instrument is not associated with confounders of the risk factor–outcome association, and that there is no influence of the genetic instrument on the outcome other than via the risk factor of interest (the latter may be violated by horizontal pleiotropy).^9,23,24^ We found some association of BMI genetic variants with smoking and income, but adjustment for these variables in the MR analyses did not alter results. The Copenhagen General Population Study participants are ethnically and geographically homogeneous, which reduces the risk of confounding due to population stratification. However, this homogeneity means that results may not generalize to other ethnic groups. Our sensitivity analyses suggest some greater influence of one of the BMI genetic variants (*FTO*) than other variants, but the results were not markedly different and our conclusions were not altered by its removal.

Another limitation of both analyses is that individuals reporting no consumption of alcohol may be a heterogeneous group of life-long abstainers and those who have previously consumed alcohol but have since become abstainers. However, the multivariable regression results suggest linear associations of increasing consumption from this group of nondrinkers and the similarity of analyses including and excluding prevalent disease cases suggest that this combination of abstainers is unlikely to have introduced major bias.

There is potential for some misclassification of liver disease cases as controls because not all cases (particularly those in the early stages) result in hospital admission or outpatient visits, and primary care records are not included in the routinely linked data used here. This lack of data would result in a diluted estimate of the association of BMI and alcohol consumption with liver disease. However, it would be expected that these patients would have elevated levels of ALT and GGT compared with individuals with no liver disease. This potential for misclassification of disease status provides a possible explanation for the differences observed when estimating the association of BMI and alcohol use with liver disease, compared with the continuous measurement of liver injury biomarkers.

## Conclusions

Taking account of both our multivariable and MR results, this study suggests that the associations between high BMI and alcohol consumption with liver injury and risk of liver disease may act independently and that these risk factors may increase biomarkers of liver injury and risk of liver disease. Interventions to reduce both of these risk factors might produce the greater benefit in terms of reducing population levels of biomarkers of liver injury than interventions aimed at either BMI or alcohol use alone. However, the current evidence is not clear whether reducing both BMI and alcohol consumption in combination will directly translate to a reduced risk in clinical liver disease; further studies are required to identify the true joint causal effect of BMI and alcohol use on liver disease.

## Notes

Supplement. **eTable 1.** ICD Codes Used for Clinical Diagnosis of Liver Disease **eTable 2.** Baseline Characteristics of All Exposure, Outcomes and Confounding Variables Amongst All Participants **eTable 3.** Distributions of Males and Females Across Observational and Genetic Factorial Groups **eTable 4.** Univariable Associations Between Body Mass Index (BMI) and Alcohol and Potential Confounders **eTable 5.** Univariable Associations Between the Weighted Allele Score for Body Mass Index (BMI) and the *ADH1B* Allele for Alcohol and Potential Confounders **eTable 6.** Univariable Associations via Multinomial Logistic Regression Between Exposures and Potential Confounders With Observational and Genetic Factorial Groups **eTable 7.** Allele Frequencies and Test for Hardy-Weinberg Equilibrium (HWE) for Each Genetic Variant **eTable 8.** Associations of Body Mass Index (BMI) and Alcohol Genetic Instruments With the Relevant Risk Factors **eTable 9.** MR Egger and Weighted Median Regression of the Association Between BMI and Each Liver Disease Amongst All Participants With Cases of Prevalent Liver Disease Excluded **eFigure 1.** Multivariable and Mendelian Randomization Individual Analysis of Body Mass Index (BMI) and Alcohol on Liver Disease Biomarkers Including All Individuals With Previously Diagnosed (Prevalent) Liver Disease **eFigure 2.** Multivariable and Mendelian Randomization Individual Analysis of Body Mass Index (BMI) and Alcohol on Liver Disease Including All Cases of Liver Disease **eFigure 3.** Multivariable Regression Associations of Measured Categories of Body Mass Index (BMI) With Biomarkers of Liver Injury and Incident Liver Disease **eFigure 4.** Multivariable Regression Associations of Alanine Amino Transferase (ALT), Γ-Glutamyltransferase (GGT), and Incident Liver Disease With Categories of Weekly Alcohol Consumption **eFigure 5.** Multivariable and Mendelian Randomization Factorial Analysis of the Joint Effects of Body Mass Index (BMI) and Alcohol on Biomarkers of Liver Injury, Including All Individuals With Previously Diagnosed (Prevalent) Liver Disease **eFigure 6.** Multivariable and Mendelian Randomization Factorial Analysis of the Joint Effects of BMI and Alcohol on Liver Disease, Including All Individuals With Previously Diagnosed (Prevalent) Liver Disease **eFigure 7.** Multivariable and Mendelian Randomization Analysis of Body Mass Index (BMI) and Liver Injury Biomarkers and Incident Cases of Liver Disease, Stratified by Self-Reported Alcohol Consumption **eFigure 8.** “Leave One Out” Analysis to Assess for Heterogeneity Between BMI Genetic Variants and Liver Injury Biomarkers and Incident Cases of Liver Disease
