# Supplementary material for: Combined Association of Body Mass Index and Alcohol Consumption With Biomarkers for Liver Injury and Incidence of Liver Disease: A Mendelian Randomization Study
Source: JAMA Netw Open. 2019 Mar 8;2(3):e190305. doi: 10.1001/jamanetworkopen.2019.0305 (PMC6484655; doi:10.1001/jamanetworkopen.2019.0305)
Supplement: Supplement. — eTable 1. ICD Codes Used for Clinical Diagnosis of Liver Disease eTable 2. Baseline Characteristics of All Exposure, Outcomes and Confounding Variables Amongst All Participants eTable 3. Distributions of Males and Females Across Observational and Genetic Factorial Groups eTable 4. Univariable Associations Between Body Mass Index (BMI) and Alcohol and Potential Confounders eTable 5. Univariable Associations Between the Weighted Allele Score for Body Mass Index (BMI) and the ADH1B Allele for Alcohol and Potential Confounders eTable 6. Univariable Associations via Multinomial Logistic Regression Between Exposures and Potential Confounders With Observational and Genetic Factorial Groups eTable 7. Allele Frequencies and Test for Hardy-Weinberg Equilibrium (HWE) for Each Genetic Variant eTable 8. Associations of Body Mass Index (BMI) and Alcohol Genetic Instruments With the Relevant Risk Factors eTable 9. MR Egger and Weighted Median Regression of the Association Between BMI and Each Liver Disease Amongst All Participants With Cases of Prevalent Liver Disease Excluded eFigure 1. Multivariable and Mendelian Randomization Individual Analysis of Body Mass Index (BMI) and Alcohol on Liver Disease Biomarkers Including All Individuals With Previously Diagnosed (Prevalent) Liver Disease eFigure 2. Multivariable and Mendelian Randomization Individual Analysis of Body Mass Index (BMI) and Alcohol on Liver Disease Including All Cases of Liver Disease eFigure 3. Multivariable Regression Associations of Measured Categories of Body Mass Index (BMI) With Biomarkers of Liver Injury and Incident Liver Disease eFigure 4. Multivariable Regression Associations of Alanine Amino Transferase (ALT), Γ-Glutamyltransferase (GGT), and Incident Liver Disease With Categories of Weekly Alcohol Consumption eFigure 5. Multivariable and Mendelian Randomization Factorial Analysis of the Joint Effects of Body Mass Index (BMI) and Alcohol on Biomarkers of Liver Injury, Including All Individuals With Previo [file jamanetwopen-2-e190305-s001.pdf]

Supplementary Online Content

Carter AR, Borges M-C, Benn M, et al. Combined association of body mass index and alcohol consumption with biomarkers for liver injury and incidence of liver disease: a mendelian randomization study. *JAMA Netw Open*. 2019;2(3):e190305. doi:10.1001/jamanetworkopen.2019.0305

eTable 1. ICD Codes Used for Clinical Diagnosis of Liver Disease
eTable 2. Baseline Characteristics of All Exposure, Outcomes and Confounding Variables Amongst All Participants
eTable 3. Distributions of Males and Females Across Observational and Genetic Factorial Groups
eTable 4. Univariable Associations Between Body Mass Index (BMI) and Alcohol and Potential Confounders
eTable 5. Univariable Associations Between the Weighted Allele Score for Body Mass Index (BMI) and the *ADH1B* Allele for Alcohol and Potential Confounders
eTable 6. Univariable Associations via Multinomial Logistic Regression Between Exposures and Potential Confounders With Observational and Genetic Factorial Groups
eTable 7. Allele Frequencies and Test for Hardy-Weinberg Equilibrium (HWE) for Each Genetic Variant
eTable 8. Associations of Body Mass Index (BMI) and Alcohol Genetic Instruments With the Relevant Risk Factors
eTable 9. MR Egger and Weighted Median Regression of the Association Between BMI and Each Liver Disease Amongst All Participants With Cases of Prevalent Liver Disease Excluded
eFigure 1. Multivariable and Mendelian Randomization Individual Analysis of Body Mass Index (BMI) and Alcohol on Liver Disease Biomarkers Including All Individuals With Previously Diagnosed (Prevalent) Liver Disease
eFigure 2. Multivariable and Mendelian Randomization Individual Analysis of Body Mass Index (BMI) and Alcohol on Liver Disease Including All Cases of Liver Disease
eFigure 3. Multivariable Regression Associations of Measured Categories of Body Mass Index (BMI) With Biomarkers of Liver Injury and Incident Liver Disease
eFigure 4. Multivariable Regression Associations of Alanine Amino Transferase (ALT), Γ-Glutamyltransferase (GGT), and Incident Liver Disease With Categories of Weekly Alcohol Consumption
eFigure 5. Multivariable and Mendelian Randomization Factorial Analysis of the Joint Effects of Body Mass Index (BMI) and Alcohol on Biomarkers of Liver Injury, Including All Individuals With Previously Diagnosed (Prevalent) Liver Disease
eFigure 6. Multivariable and Mendelian Randomization Factorial Analysis of the Joint Effects of BMI and Alcohol on Liver Disease, Including All Individuals With Previously Diagnosed (Prevalent) Liver Disease
eFigure 7. Multivariable and Mendelian Randomization Analysis of Body Mass Index (BMI) and Liver Injury Biomarkers and Incident Cases of Liver Disease, Stratified by Self-Reported Alcohol Consumption
eFigure 8. “Leave One Out” Analysis to Assess for Heterogeneity Between BMI Genetic Variants and Liver Injury Biomarkers and Incident Cases of Liver Disease

This supplementary material has been provided by the authors to give readers additional information about their work.

© 2019 Carter AR et al. *JAMA Network Open*.

eTable 1. ICD Codes Used for Clinical Diagnosis of Liver Disease

	ICD 8	ICD10
Chronic liver disease and cirrhosis	571.00-571.7	K70.0-K70.9
Other and Unspecified chronic liver disease without mention of alcohol	571.8, 571.9	K74.6, K75.8
Hepatic fibrosis		K74.0
Malignant neoplasms of the liver and other malignancies	155.09-155.89	C22, R18
Acute and subacute necrosis of the liver	570.00-570.99	
Chronic passive congestion of the liver – unspecified disorder of the liver	573.00-573.9	K75.9
Abdominal swelling	785.19-785.39	
Other disease of the liver		K76.0, K76.9

© 2019 Carter AR et al. *JAMA Network Open*.

eTable 2. Baseline Characteristics of All Exposure, Outcomes and Confounding Variables Amongst All Participants

Characteristic	All Participants [%]	Female [%]	Male [%]
Sex	98 643		
Male	44 344 [55.05]		
Female	54 299 [44.95]		
Age years	98 643	54 299	44 344
20-25	796 [0.81]	458 [0.84]	338 [0.76]
26-30	1020 [1.03]	554 [1.02]	466 [1.05]
31-35	1822 [1.85]	1055 [1.94]	767 [1.73]
36-40	3096 [3.14]	1803 [3.32]	1293 [2.92]
41-45	10 177 [10.32]	5866 [10.80]	4311 [9.72]
46-50	12 564 [12.74]	7102 [13.08]	5462 [12.32]
51-55	12 324 [12.49]	6732 [12.40]	5592 [12.61]
56-60	12 428 [12.60]	6924 [12.75]	5504 [12.41]
61-65	13 844 [14.03]	7639 [14.07]	6205 [13.99]
66-70	11 956 [12.12]	6452 [11.88]	5504 [12.41]
71-75	8547 [8.66]	4485 [8.26]	4062 [9.16]
76-80	5534 [5.61]	2868 [5.28]	2666 [6.01]
>81	4535 [4.60]	2361 [4.35]	2174 [4.90]
BMI	98 307	54 031	44 276
Underweight	696 [0.71]	598 [1.11]	98 [0.22]
Normal	42 140 [42.87]	27 656 [51.19]	14 484 [32.71]
Overweight	39 474 [40.15]	17 483 [32.36]	21 991 [49.67]
Obese	15 997 [16.27]	8294 [15.35]	7703 [17.40]
Weekly Alcohol Consumption	98 579	54 260	44 319
None	9308 [9.44]	6745 [12.43]	2563 [5.78]
1-7 Units	38 093 [38.64]	25 347 [46.71]	12 746 [28.76]
8-14 Units	25 570 [25.94]	13 814 [25.46]	11 756 [26.53]
15-21 Units	13 517 [13.71]	5592 [10.31]	7925 [17.88]
22-34 Units	9105 [9.24]	2342 [4.32]	6763 [15.26]
>35 Units	2986 [3.03]	420 [0.77]	2566 [5.79]
Liver Disease	98 643	54 299	44 344
None	97 238 [98.58]	53 570 [98.78]	43 668 [98.48]
All Cases	1405 [1.42]	729 [1.34]	676 [1.52]
Removing Prevalent cases	626 [0.63]	325 [0.60]	301 [0.68]
Education	98 312	54 119	44 193
<10 years	25 275 [25.71]	13 134 [24.27]	12 141 [27.47]
10-13 years	54 937 [55.88]	30 871 [57.04]	24 066 [54.46]
>13 years	18 100 [18.41]	10 114 [18.69]	7986 [18.07]
Smoking	98 038	54 132	43 906
Never Smoker	41 674 [42.51]	24 472 [45.21]	17 202 [39.18]
Former Smoker	39 061 [39.84]	20 514 [37.90]	18 547 [42.24]
Current Smoker	17 303 [17.65]	9146 [16.90]	8157 [18.58]

© 2019 Carter AR et al. *JAMA Network Open.*

Income	97 379	53 391	43 988
<100.000kr	1506 [1.55]	973 [1.82]	533 [1.21]
100.000 – 400.000kr	34 225 [35.15]	20 894 [39.13]	13 331 [30.31]
400.000-600.000kr	38 150 [39.18]	19 806 [37.10]	18 344 [41.70]
>600.000kr	23 498 [24.13]	11 718 [21.95]	11 780 [26.78]
Leisure Time Physical Activity	97 828	53 777	44 051
<2 hours per week	6233 [6.4]	3310 [6.16]	2923 [6.64]
2-4 hours per week	41 777 [42.7]	25 209 [46.88]	16 568 [37.61]
Moderate >4 hours per week/Heavy 2-4 hours per week	43 416 [44.4]	23 197 [43.14]	20 219 [45.90]
>4 hours heavy activity	6402 [6.5]	2061 [3.83]	4341 [9.85]
FTO	96 770	53 225	43 545
Homozygous [decreasing]	34 431 [35.58]	19 010 [35.72]	15 421 [35.41]
Heterozygous	46 869 [48.43]	25 691 [48.27]	21 178 [48.63]
Homozygous [increasing]	15 470 [15.99]	8524 [16.02]	6946 [15.95]
TMEM18	97 443	53 598	43 845
Homozygous [decreasing]	2712 [2.78]	1481 [2.76]	1231 [2.81]
Heterozygous	27 438 [28.16]	15 128 [28.22]	12 310 [28.08]
Homozygous [increasing]	67 293 [69.06]	36 989 [69.01]	30 304 [69.12]
MC4R	97 465	53 608	43 857
Homozygous [decreasing]	55 165 [56.60]	30 297 [56.52]	24 868 [56.70]
Heterozygous	36 325 [37.27]	20 002 [37.31]	16 323 [37.22]
Homozygous [increasing]	5975 [6.13]	3309 [6.17]	2666 [6.08]
BDNF	97 530	53 633	43 897
Homozygous [decreasing]	5163 [5.29]	2795 [5.21]	2368 [5.39]
Heterozygous	34 495 [35.37]	18 888 [35.22]	15 607 [35.55]
Homozygous [increasing]	57 872 [59.34]	31 950 [59.57]	25 992 [59.05]
GNPDA2	97 904	53 850	44 054
Homozygous [decreasing]	34 508 [35.25]	19 052 [35.38]	15 456 [35.08]
Heterozygous	47 323 [48.34]	25 995 [48.27]	21 328 [48.41]
Homozygous [increasing]	16 073 [16.42]	8803 [16.35]	770 [16.50]
ADH1B	97 925	53 869	44 056
Homozygous [Alcohol decreasing]	43 [0.04]	22 [0.04]	21 [0.05]
Heterozygous	3966 [4.05]	2131 [3.96]	1835 [4.17]
Homozygous [Alcohol Increasing]	93 916 [95.91]	51 716 [16.50]	42 200 [95.79]

© 2019 Carter AR et al. *JAMA Network Open.*

eTable 3. Distributions of Males and Females Across Observational and Genetic Factorial Groups

		Low BMI & Low Alcohol [%]	Low BMI & High Alcohol [%]	High BMI & Low Alcohol [%]	High BMI & High Alcohol [%]
Observational	Female	15 830 [31.33]	10 185 [20.16]	16 590 [32.84]	7918[15.67]
	Male	7843 [18.79]	12 384 [29.68]	8367 [20.05]	13 138 [31.48]
Genetic	Female	1045 [2.07]	24 583 [48.66]	986 [1.95]	23 909 [47.32]
	Male	884 [2.12]	20 367 [48.80]	862 [2.07]	19 619 [47.01]

© 2019 Carter AR et al. *JAMA Network Open.*

eTable 4. Univariable Associations Between Body Mass Index (BMI) and Alcohol and Potential Confounders.

Covariate	Mean difference in measured BMI (95% CI)	Mean difference in self-reported weekly alcohol consumption (95% CI)	Odds ratio of incident liver disease (95% CI)
Age years			
<30	Reference	Reference	Reference
30-39	0.74 (0.51, 0.98)	-0.80 (-1.36, -0.24)	5.06 (1.20, 21.28)
40-49	1.30 (1.09, 1.52)	1.86 (1.36, 2.35)	7.05 (1.75, 28.44)
50-59	1.89 (1.68, 2.10)	4.82 (4.32, 5.31)	11.86 (2.95, 47.66)
60-69	2.26 (2.05, 2.47)	6.20 (5.70, 6.70)	15.52 (3.86, 62.29)
70-79	2.28 (1.33, 1.81)	5.31 (4.80, 5.82)	14.54 (3.61, 58.57)
80+	1.57 (1.33, 1.81)	3.97 (3.40, 4.54)	17.28 (4.23, 70.12)
Sex			
Female	Reference	Reference	Reference
Male	1.21 (1.16, 1.27)	6.23 (6.10, 6.36)	1.17 (1.00, 1.38)
Education			
<10 years	Reference	Reference	Reference
10-13 years	-1.37 (-1.43, -1.30)	-0.12 (-0.28, 0.03)	0.46 (0.38, 0.55)
>13 years	-1.79 (-1.88. -1.71)	-1.55 (-1.75, -1.35)	0.41 (0.31, 0.53)
Smoking			
Never	Reference	Reference	Reference
Ever	0.36 (0.30, 0.41)	2.90 (2.77, 3.03)	1.68 (1.40, 2.00)
Income			
<100.000 kr	Reference	Reference	Reference
100.000kr – 400.000kr	0.17 (-0.06, 0.40)	1.99 (1.44, 2.55)	0.88 (0.52, 1.48)
400.000kr – 600.000 kr	-0.30 (-0.52, -0.06)	2.52 (1.96, 3.07)	0.44 (0.26, 0.74)
>600.000 kr	-1.08 (-1.31, -0.85)	2.57 (2.01, 3.13)	0.31 (0.18, 0.55)
Physical Activity			
0-4 hours weekly	Reference	Reference	Reference
>4 hours weekly	-1.21 (-1.27, -1.16)	0.71 (0.58, 0.84)	0.70 (0.60, 0.83)

© 2019 Carter AR et al. *JAMA Network Open.*

eTable 5. Univariable associations between the weighted allele score for body mass index (BMI) and the *ADH1B* allele for alcohol and potential confounders

Covariate	Mean difference increase in BMI according to BMI genotype (95% CI)	Mean difference in weekly alcohol consumption according to alcohol genotype (95% CI)
BMI		
Normal/Underweight	Reference	Reference
Overweight	0.006 (0.005, 0.007)	0.002 (-0.001, 0.005)
Obese	0.014 (0.013, 0.016)	0.006 (0.002, 0.009)
Weekly alcohol consumption		
0 units	Reference	Reference
1-14 units	-0.002 (-0.003, 0.0002)	0.014 (0.009, 0.018)
>15 units	-0.001 (-0.003, 0.0007)	0.024 (0.019, 0.029)
Age years		
<30	Reference	Reference
30-39	.0002 (-0.004, 0.005)	-0.010 (-0.021, 0.002)
40-49	-0.003 (-0.007, 0.001)	-0.013 (-0.023, -0.003)
50-59	-0.003 (-0.007, 0.001)	-0.013 (-0.023, -0.023)
60-69	-0.002 (-0.006, 0.002)	-0.010 (-0.020, -0.00003)
70-79	-0.002 (-0.006, 0.002)	-0.014 (-0.025, -0.004)
80+	-0.003 (-0.007, 0.002)	-0.014 (-0.025, -0.002)
Sex		
Female	Reference	Reference
Male	-.00003 (-0.001, 0.001)	-0.002 (-0.004, 0.001)
Education		
<10 years	Reference	Reference
10-13 years	-0.001 (-0.002, 0.00004)	-0.003 (-0.006, 0.001)
>13 years	-0.0004 (-0.002, 0.001)	-0.003 (-0.007, 0.001)
Smoking		
Never	Reference	Reference
Ever	0.002 (0.0006, 0.003)	-0.003 (-0.005, -0.0001)
Income		
<100.000 kr	Reference	Reference
100.000kr – 400.000kr	-0.003 (-0.007, 0.002)	-0.010 (-0.021, 0.001)
400.000kr – 600.000 kr	-0.003 (-0.007, 0.001)	-0.010 (-0.021, 0.001)
>600.000 kr	-0.005 (-0.010, -0.001)	-0.10 (-0.021, 0.001)
Physical Activity		
0-4 hours weekly	Reference	Reference
>4 hours weekly	0.001 (-0.001, 0.002)	-0.002 (-0.005, 0.001)

© 2019 Carter AR et al. *JAMA Network Open.*

eTable 6. Univariable Associations via Multinomial Logistic Regression Between Exposures and Potential Confounders With Observational and Genetic Factorial Groups

Covariate	Log odds ratio for observational factorial groups (95% CI)				Log odds ratio for genetic factorial groups (95% CI)			
	**Low BMI & Low Alcohol**	**Low BMI & High Alcohol**	**High BMI & Low Alcohol**	**High BMI & High Alcohol**	**Low BMI & Low Alcohol**	**Low BMI & High Alcohol**	**High BMI & Low Alcohol**	**High BMI & High Alcohol**
BMI	Reference	0.67 (0.61, 0.72)	4.99 (4.93, 5.06)	4.95 (4.88, 5.01)	Reference	0.07 (0.0001, 0.13)	0.15 (0.06, 0.24)	0.21 (0.15, 0.28)
Weekly alcohol consumption	Reference	8.58 (7.89, 9.27)	-0.31 (-0.36, -0.27)	8.71 (8.02, 9.40)	Reference	0.26 (0.18, 0.34)	-0.06 (-0.17, 0.06)	0.26 (0.18, 0.34)
Age years								
<30	Reference				Reference			
30-39		-0.37 (-0.55, -0.19)	-0.03 (-0.16, 0.10)	-0.33 (-0.51, -0.15)		-0.22 (-0.67, 0.22)	0.17 (-0.47 0.80)	-0.17 (-0.62, 0.28)
40-49		0.44 (0.28, 0.59)	0.05 (-0.07, 0.17)	0.39 (0.23, 0.55)		-0.38 (-0.78, 0.19)	-0.004 (-0.58, 0.57)	-0.37 (-0.77, 0.04)
50-59		1.09 (0.94, 1.25)	0.11 (-0.004, 0.23)	1.06 (0.90, 1.21)		-0.29 (-0.69, 0.11)	0.14 (-0.44, 0.71)	-0.29 (-0.69, 0.11)
60-69		1.36 (1.21, 1.52)	0.20 (0.08, 0.32)	1.39 (1.23, 1.55)		-0.28 (-0.69, 0.11)	0.06 (-0.51, 0.64)	-0.26 (-0.66, 0.15)
70-79		1.27 (1.11, 1.43)	0.24 (0.11, 0.36)	1.30 (1.13, 1.46)		-0.35 (-0.76, 0.06)	0.14 (-0.45, 0.71)	-0.31 (-0.72, 0.10)
80+		0.91 (0.74, 1.09)	0.18 (0.04, 0.32)	1.07 (0.90, 1.25)		-0.38 (-0.82, 0.07)	0.05 (-0.58, 0.69)	-0.35 (-0.80, 0.09)

© 2019 Carter AR et al. *JAMA Network Open.*

Sex								
Female	Reference				Reference			
Male		0.90 (0.86, 0.93)	0.02 (-0.02, 0.05)	1.21 (1.17, 1.25)		-0.02 (-0.11, 0.07)	0.03 (-0.10, 0.16)	-0.03 (-0.13, 0.06)
Education								
<10 years	Reference				Reference			
10-13 years		0.01 (-0.04, 0.06)	-0.46 (-0.50, -0.41)	-0.46 (-0.50, -0.41)		-0.07 (-0.19, 0.04)	-0.04 (-0.20, 0.11)	-0.08 (-0.20, 0.03)
>13 years		-0.32 (-0.38, -0.26)	-0.58 (-0.63, -0.52)	-0.81 (-0.87, -0.75)		-0.07 (-0.21, 0.07)	-0.02 (-0.22, 0.17)	-0.07 (-0.21, 0.07)
Smoking								
Never	Reference				Reference			
Ever		0.52 (0.48, 0.55)	0.17 (0.13, 0.20)	0.60 (0.57, 0.64)		-0.51 (-0.14, 0.04)0	0.08 (-0.05, 0.21)	-0.02 (-0.11, 0.08)
Income								
<100.000 kr	Reference				Reference			
100.000kr – 400.000kr		0.41 (0.24, 0.58)	-0.04 (-0.17, 0.10)	0.41 (0.24, 0.57)		-0.15 (-0.55, 0.26)	0.20 (-0.41, 0.82)	-0.19 (-0.60, 0.21)
400.000kr – 600.000 kr		0.51 (0.35, 0.68)	-0.21 (-0.35, -0.07)	0.41 (0.25, 0.57)		-0.06 (-0.46, 0.35)	0.40 (-0.21, 1.01)	-0.11 (--0.52, 0.30)
>600.000 kr		0.54 (0.37, 0.71)	-0.55 (-0.69, -0.41)	0.20 (0.03, 0.36)		-0.12 (-0.53, 0.29)	0.23 (-0.39, 0.85)	-0.20 (-0.61, 0.21)
Physical Activity								
0-4 hours weekly	Reference				Reference			
>4 hours weekly		0.15 (0.11, 0.18)	-0.50 (-0.54, -0.47)	-0.58 (-0.32, -0.24)		-0.004 (-0.10, 0.09)	0.09 (-0.04, 0.21)	-0.01 (-0.10, 0.08)

BMI = Body Mass Index CI = Confidence Interval

© 2019 Carter AR et al. *JAMA Network Open.*

eTable 7. Allele Frequencies and Test for Hardy-Weinberg Equilibrium (HWE) for Each Genetic Variant

Genetic variant	Minor Allele Frequency	HWE P value
FTO	0.36	0.06
TMEM18	0.28	0.17
MC4R	*0.37*	*0.79*
BDNF	0.35	0.98
GNPDA2	0.35	0.63
ADH1B	0.04	0.68

© 2019 Carter AR et al. *JAMA Network Open.*

**eTable 8. Associations of Body Mass Index (BMI) and Alcohol Genetic Instruments With the Relevant Risk Factors (First-stage regression analyses relevant to instrument strength
n=92 255)**

Genetic Variant	Per risk allele increase in BMI SD (95% CI)	F statistic	R-squared
FTO	0.08 [0.07, 0.08]	251	0.0027
TMEM18	0.05 [0.03, 0.06]	56	0.0006
MC4R	0.05 [0.04, 0.06]	82	0.0009
BDNF	0.03 [0.02, 0.05]	38	0.0004
GNPDA2	0.03 [0.02, 0.04]	34	0.0004
Weighted Allele	0.86 [0.78, 0.94]	453	0.0049
	**Per risk allele increase in alcohol consumption (units per week)**	**F statistic**	**R-squared**
ADH1B	1.81 [1.48,2.14]	117.4	0.0013

© 2019 Carter AR et al. *JAMA Network Open.*

eTable 9. MR Egger and Weighted Median Regression of the Association Between BMI and Each Liver Disease Amongst All Participants With Cases of Prevalent Liver Disease Excluded

MR Egger estimates in all participants			
	ALT Difference in mean % [95% CI]	GGT Difference in mean % [95% CI]	Liver Disease OR [95% CI]
MR Egger estimate	42.49 [10.21,74.78]	26.45 [-87.35,66.15]	1.23 [-6.79,9.24]
MR Egger constant	-73.26 [-45.26, 1.28]	-84.50 [-50.32,18.68]	-0.59 [-7.63,9.24]
Weighted Median	11.95 [7.07,16.84]	9.04 [3.82,14.27]	1.00 [0.99,1.01]

CI = confidence interval; OR = Odds ratio

© 2019 Carter AR et al. *JAMA Network Open.*

eFigure 1. Multivariable and Mendelian Randomization Individual Analysis of Body Mass Index (BMI) and Alcohol on Liver Disease Biomarkers Including All Individuals With Previously Diagnosed (Prevalent) Liver Disease

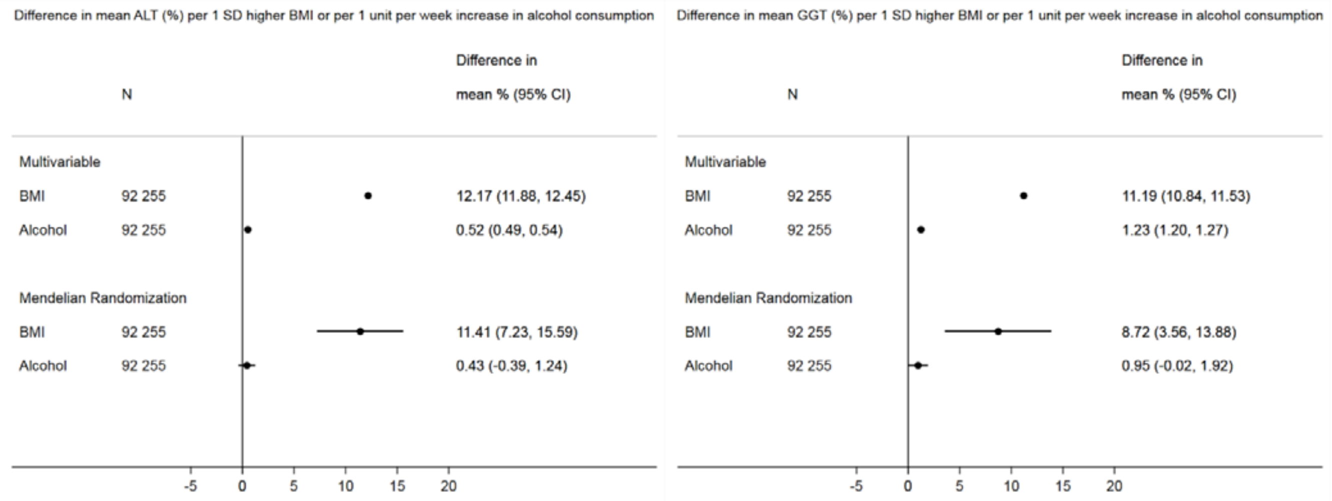

BMI measured as age and sex standardised units. Alcohol as units of alcohol consumed per week, where one unit is equivalent to 12g of alcohol. MV = Multivariable analysis, adjusted for Models adjusted for age, sex, smoking, education, income and physical activity. MR= Mendelian Randomization, using weighted allele score for BMI. CI = confidence interval.

© 2019 Carter AR et al. *JAMA Network Open.*

eFigure 2. Multivariable and Mendelian Randomization Individual Analysis of Body Mass Index (BMI) and Alcohol on Liver Disease Including All Cases of Liver Disease

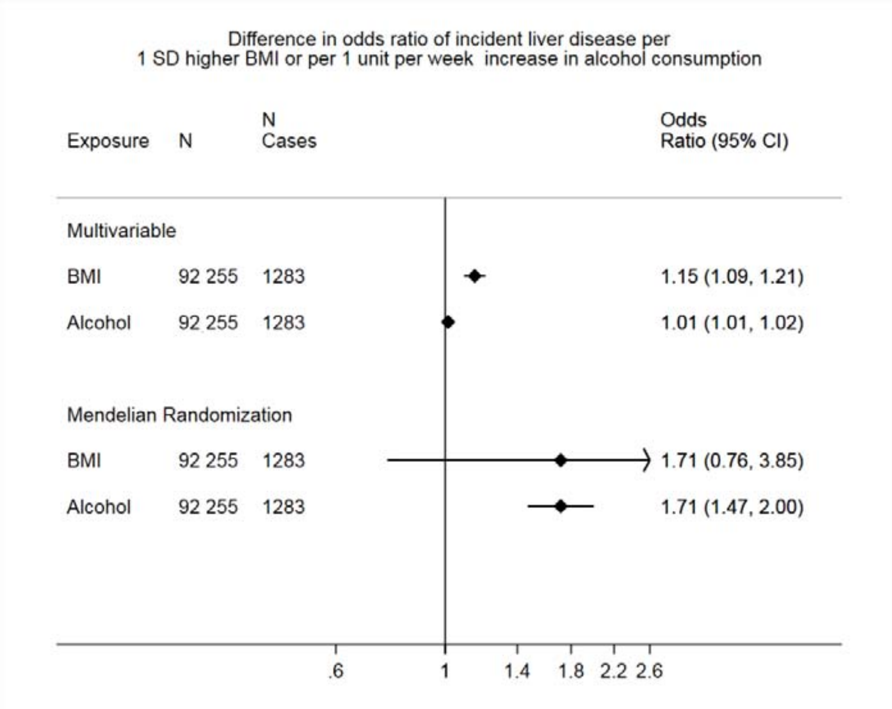

BMI measured as age and sex standardised units. Alcohol as units of alcohol consumed per week, where one unit is equivalent to 12g of alcohol. MV = Multivariable analysis, adjusted for Models adjusted for age, sex, smoking, education, income and physical activity. MR= Mendelian Randomization, using weighted allele score for BMI. CI = confidence interval.

© 2019 Carter AR et al. *JAMA Network Open.*

eFigure 3. Multivariable Regression Associations of Measured Categories of Body Mass Index (BMI) With Biomarkers of Liver Injury and Incident Liver Disease

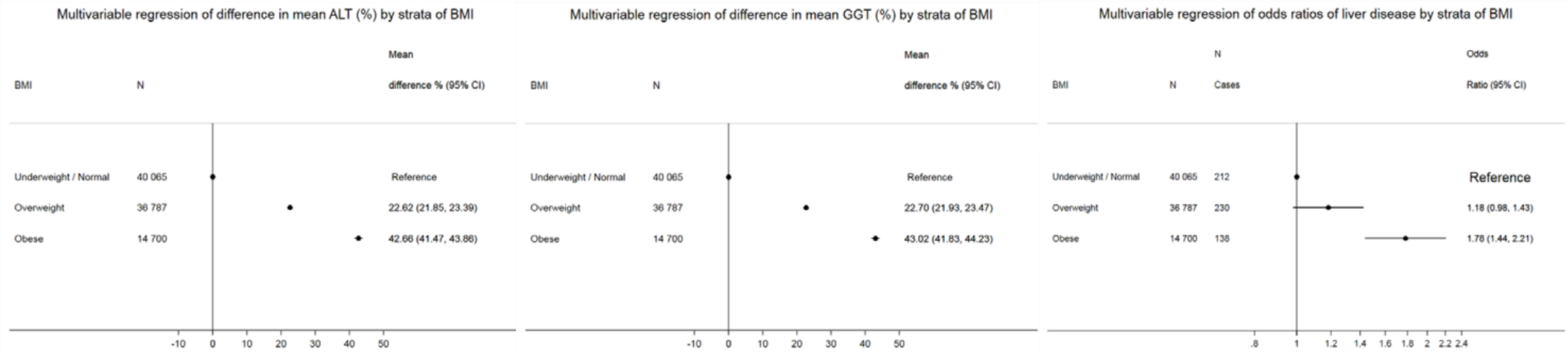

Models adjusted for age, sex, smoking, education, income and physical activity. CI = confidence

© 2019 Carter AR et al. *JAMA Network Open.*

eFigure 4. Multivariable Regression Associations of Alanine Amino Transferase (ALT) (A), Γ-Glutamyltransferase (GGT) (B), and Incident Liver Disease (C) With Categories of Weekly Alcohol Consumption

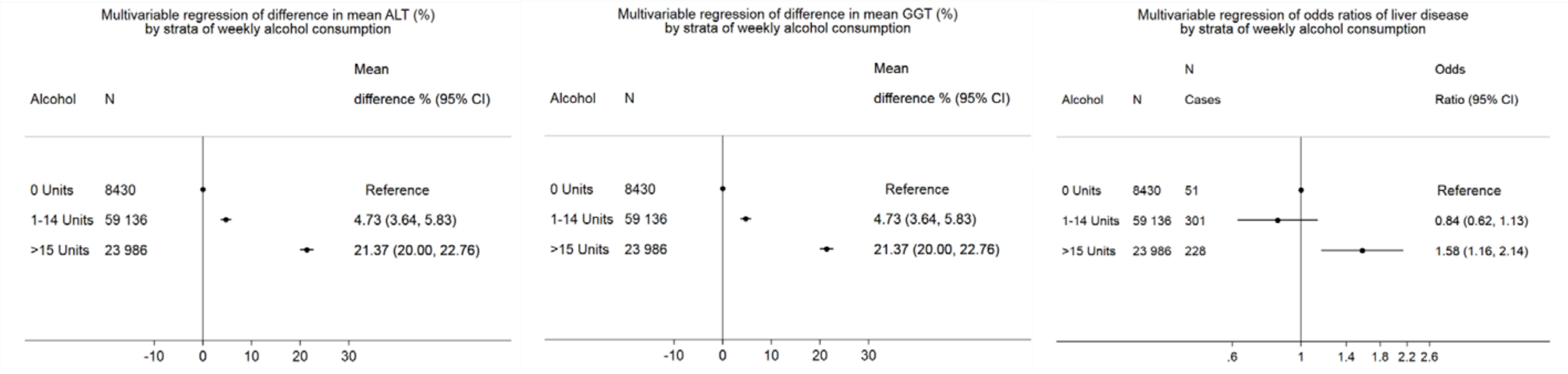

Alcohol as categorical units of alcohol consumed per week, where one unit is equivalent to 12g of alcohol. Models adjusted for age, sex, smoking, education, income and physical activity. CI = confidence interval.

© 2019 Carter AR et al. *JAMA Network Open.*

eFigure 5. Multivariable and Mendelian Randomization Factorial Analysis of the Joint Effects of Body Mass Index (BMI) and Alcohol on Biomarkers of Liver Injury, Including All Individuals With Previously Diagnosed (Prevalent) Liver Disease

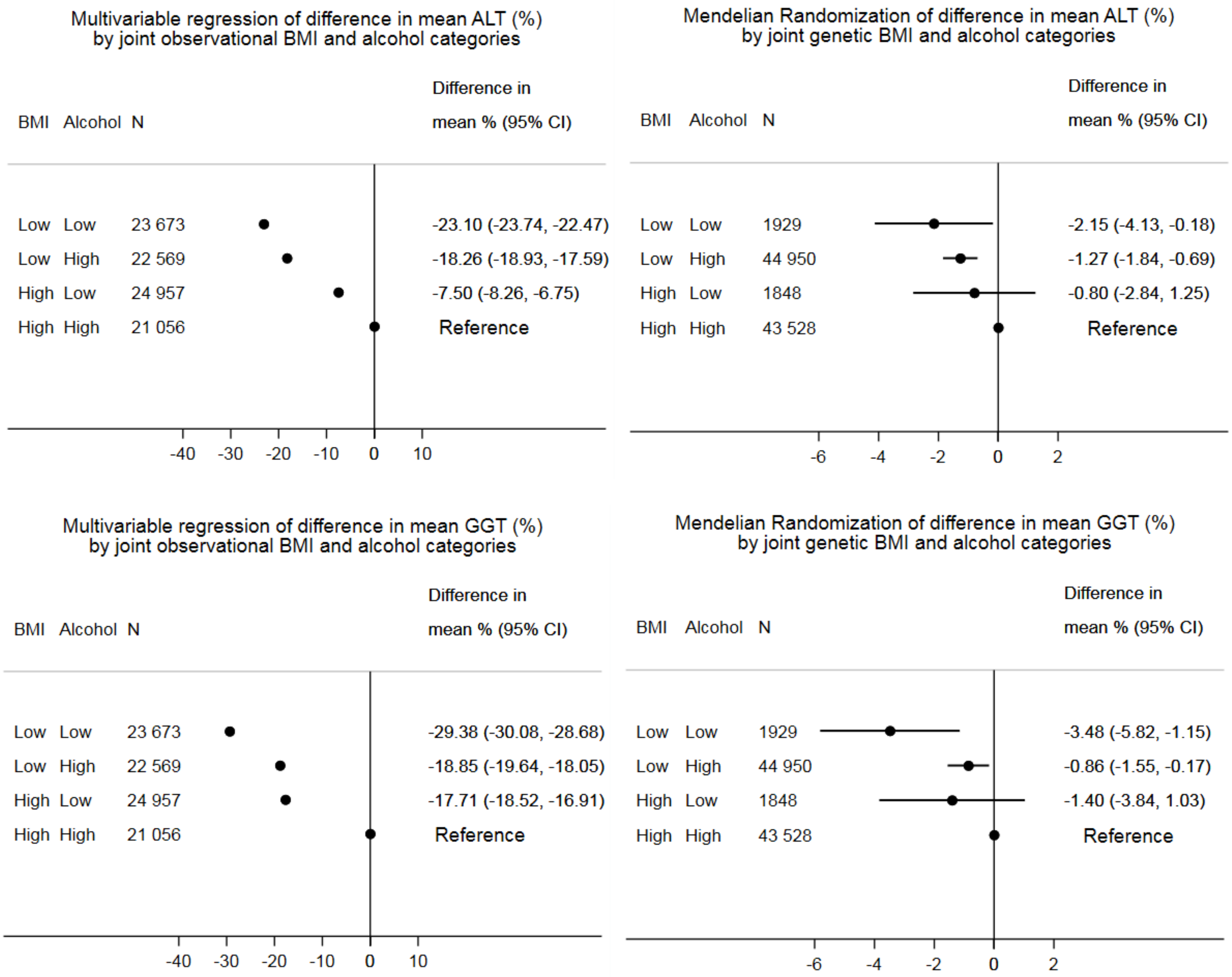

Low vs high BMI = 1.49 km/m^2 difference in MV analyses and 0.51 km/m^2 in MR analyses. Low vs high alcohol = 14.68 units per week difference in MV analyses and 1.78 units per week difference in MR analyses, where one unit of alcohol is equivalent to 12g. MV = Multivariable analysis, adjusted for age, sex, smoking, education, income and physical activity. MR= Mendelian Randomization CI = confidence interval. ALT = Alanine Amino Transferase. GGT = γ-glutamyltransferase

© 2019 Carter AR et al. *JAMA Network Open.*

eFigure 6. Multivariable and Mendelian Randomization Factorial Analysis of the Joint Effects of BMI and Alcohol on Liver Disease, Including All Individuals With Previously Diagnosed (Prevalent) Liver Disease

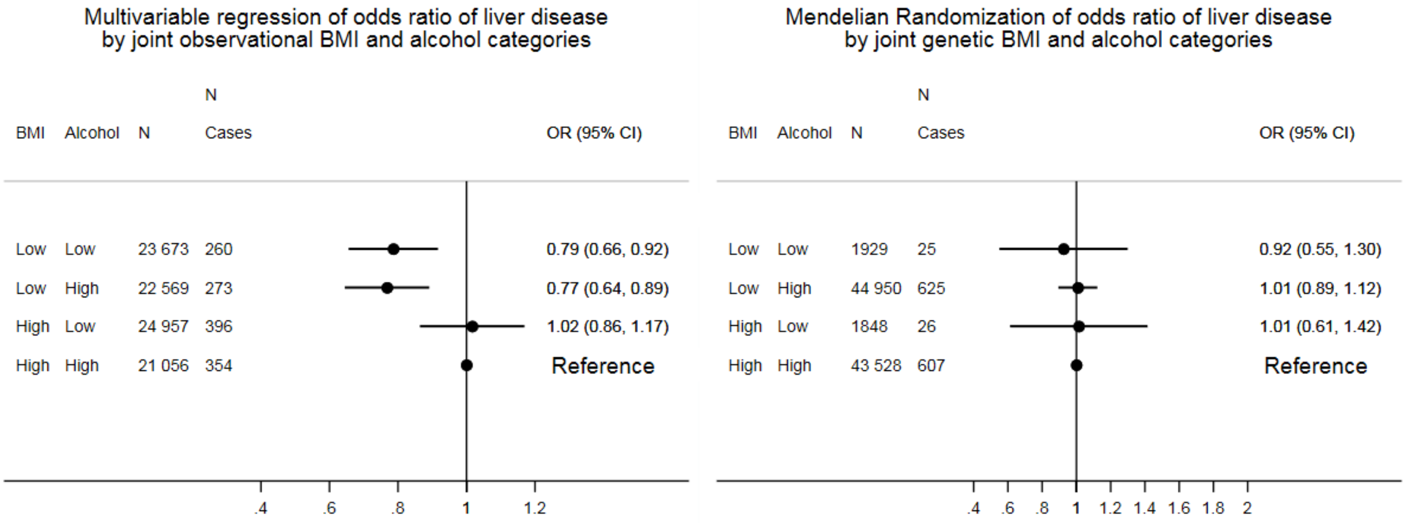

Low vs high BMI = 1.49 km/m^2 difference in MV analyses and 0.51 km/m^2 in MR analyses. Low vs high alcohol = 14.68 units per week difference in MV analyses and 1.78 units per week difference in MR analyses, where one unit of alcohol is equivalent to 12g. MV = Multivariable analysis, adjusted for age, sex, smoking, education, income and physical activity. MR= Mendelian Randomization CI = confidence interval.

© 2019 Carter AR et al. *JAMA Network Open.*

eFigure 7. Multivariable and Mendelian Randomization Analysis of Body Mass Index (BMI) and Liver Injury Biomarkers and Incident Cases of Liver Disease, Stratified by Self-Reported Alcohol Consumption

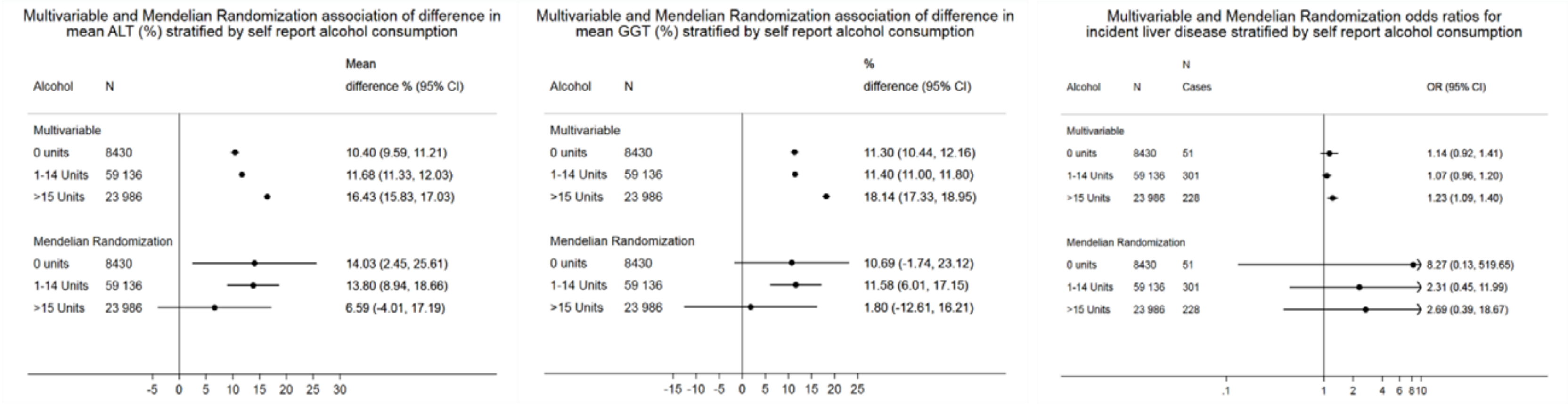

BMI as age and sex standardised units in MV analyses and as a weighted allele score in MR analyses. Alcohol as categorical units of alcohol consumed per week, where one unit is equivalent to 12g of alcohol. MV= Multivariable models adjusted for age, sex, smoking, education, income and physical activity. CI = confidence interval

© 2019 Carter AR et al. *JAMA Network Open.*

eFigure 8. "Leave One Out" Analysis to Assess for Heterogeneity Between BMI Genetic Variants and Liver Injury Biomarkers and Incident Cases of Liver Disease

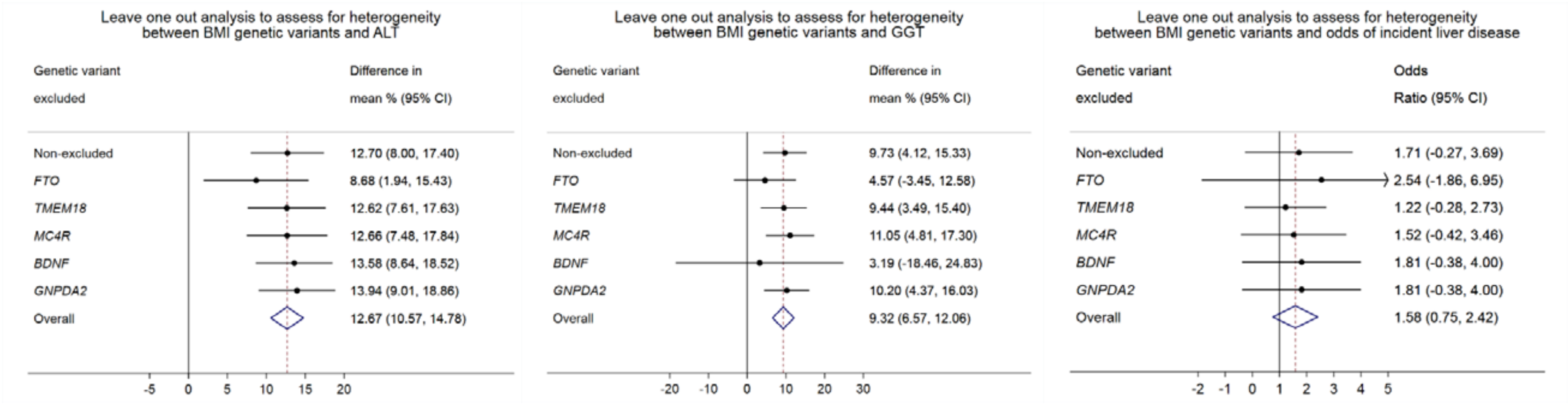

© 2019 Carter AR et al. *JAMA Network Open.*
